# Characteristics and Mechanism of Hematite Dissolution and Release on Arsenic Migration in Heterogeneous Materials

**DOI:** 10.3390/toxics12090687

**Published:** 2024-09-23

**Authors:** Zheying Li, Huimei Shan, Wanyue Rong, Zhicheng Zhao, Kexin Ma, Sanxi Peng, Song Wei

**Affiliations:** 1 Guangxi Key Laboratory of Environmental Pollution Control Theory and Technology, Guilin University of Technology, Guilin 541004, China; 15837571133@163.com (Z.L.); ke-xma2002@163.com (K.M.); 2 Collaborative Innovation Center for Water Pollution Control and Water Safety in Karst Area, Guilin University of Technology, Guilin 541004, China; 3 College of Earth Sciences, Guilin University of Technology, Guilin 541004, China

**Keywords:** arsenic, heterogeneity, hematite, migration, dissolution

## Abstract

The migration of arsenic in groundwater is influenced by the heterogeneity of the medium, and the presence of iron minerals adds complexity and uncertainty to this effect. In this study, a stratified heterogeneous sand column with an embedded hematite lens at the coarse-to-medium sand interface was designed. We introduced an arsenic-laden solution and controlled groundwater flow to investigate the spatiotemporal characteristics of arsenic migration and the impact of hematite dissolution. The results showed that the medium structure significantly influenced the arsenic migration and distribution within the lens-containing sand column. The clay layers directed the lateral migration of arsenic, and the arsenic concentrations in deeper layers were up to seven times greater than those on the surface. The extraction experiments of solid-phase arsenic revealed that the main adsorption modes on quartz sand surfaces were the specific adsorption (F2) and adsorption on weakly crystalline iron–aluminum oxides (F3), correlating to the specific and colloidal adsorption modes, respectively. Monitoring the total iron ions (Fe(aq)) revealed rapid increases within the first 14 days, reaching a maximum on day 15, and then gradually declining; these results indicate that hematite did not continuously dissolve. This study can aid in the prevention and control of arsenic contamination in groundwater.

## 1. Introduction

Arsenic (As) is found in groundwater worldwide [1,2]. The World Health Organization stipulates that the As concentration in drinking water should not exceed 10 μg/L [3]. Drinking groundwater contaminated with As can cause cancer and diseases of the lungs, liver, kidneys, and skin [4,5]. As often occurs in groundwater, and its migration and distribution are affected by iron minerals, medium heterogeneity, and human activities [6,7,8]. Recent studies have shown that the heterogeneity of the medium affects the temporal and spatial variations in groundwater arsenic concentrations [9,10]. Hematite is a common natural mineral and adds complexity to arsenic migration in heterogeneous media. Hematite dissolves into hydrated iron oxides that adsorb arsenic [11] and directly adsorb arsenic through inner-sphere complexation [12]. Therefore, the effects of the medium heterogeneity on As migration and transformation have received increasing attention.

Currently, the effect of medium heterogeneity on As migration is still unclear. During the groundwater flow process, the migration rate of arsenic may be influenced by mineral adsorption, particularly the adsorption of minerals such as clays and oxides/hydroxides of iron, aluminum, and manganese [13]. Due to the varying distribution of these minerals in sediments, they can retain arsenic to different extents, thereby affecting the migration and distribution of arsenic [14]. Duan (2020) [15] combined experimental work with sand columns and data simulations to determine that pumping enhances groundwater flow and arsenic migration along preferred pathways. They discovered that both physical and chemical heterogeneities jointly influenced these processes. Their results showed that physical heterogeneity alone could not fully explain the findings, highlighting the significant impact of medium heterogeneity on arsenic migration. Duan (2022) [16] conducted an in-depth study on the desorption of arsenic by using a heterogeneous sand tank and competitive adsorption ions. They reported that arsenic desorption was driven not only by the competitive adsorption of phosphate irons (PO_4_^3−^), but also occurred in the preferential flow channels. This study highlighted the significant role of medium heterogeneity in influencing arsenic migration and transformation. Although the heterogeneity and competitive adsorption of ions have been proven to be very important, the impact of natural hematite on arsenic migration in heterogeneous systems has yet to receive adequate attention.

The characteristics of arsenic adsorption by iron minerals have been extensively studied. For example, iron and aluminum minerals affect arsenic adsorption and desorption through dissolution processes [17,18,19]. Specifically, hematite, which is a typical iron mineral in high-arsenic areas, not only adsorbs arsenic on its surface or within its crystal structure but also dissolves due to river water infiltration, leading to the release of arsenic. This process further influences the speciation of arsenic [20,21]. In their study of West Bengal’s groundwater, Nath (2005) [22] reported that arsenic-rich areas had sandy aquifers mixed with fine sediments rich in arsenic and iron. These findings indicated that iron oxides in groundwater were crucial in controlling arsenic migration in shallow aquifers. Fendorf (2010) [23] noted from their examination of groundwater wells in the Himalayan region that the reduction and dissolution of iron oxides such as hematite not only released arsenic but also converted As(V) to the more mobile As(III). This process was influenced by groundwater movement and altered the concentration and distribution of arsenic. Due to the crucial role of hematite in arsenic migration, investigating the impacts of medium heterogeneity and the dissolution of hematite on arsenic mobility is essential.

Based on the aforementioned scientific issues, in this study, a heterogeneous sand column was designed, different-sized quartz sand was layered in the reactor, strips of clay were embedded to simulate lenses, and hematite spheres were incorporated at the coarse-to-medium sand interface. An arsenic-laden solution was introduced, and the groundwater flow was controlled by pumping to investigate the spatiotemporal characteristics of arsenic migration and the impact of hematite dissolution. Here, characterization analysis was combined with the changes in the iron concentration to determine the mechanisms involved. The aims of this study are (1) to assess the impact and characteristics of pumping and hematite on arsenic migration and (2) to explore the dissolution and release patterns of hematite under the influence of pumping. This study deepens our understanding of arsenic migration and transformation in contaminated areas and can provide a scientific basis for arsenic pollution control.

## 2. Experimental Materials and Analytical Methods

### 2.1. Sand Trough Structure and Operation Mode

**Figure 1:**
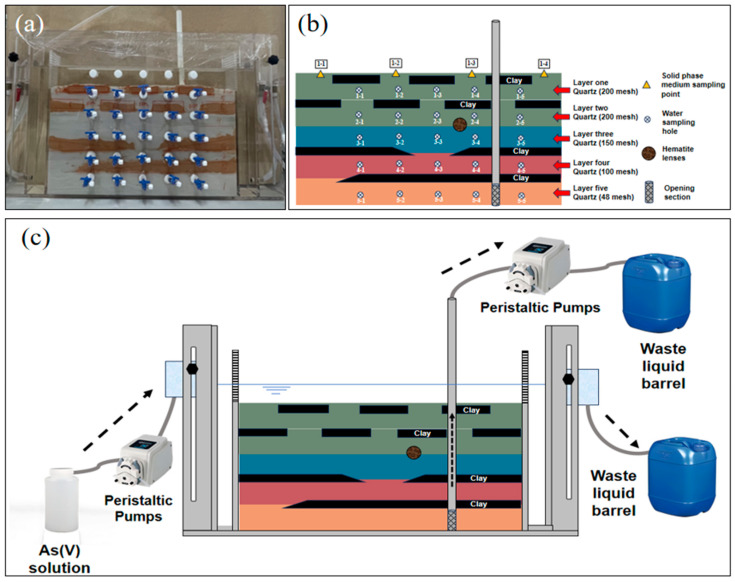
Experimental device. (**a**) Actual image; (**b**) medium structure; (**c**) device operation.

A sand tank experimental device is used to characterize the migration and transformation of pollutants in the aquifer and includes the tank body, fixed rod, PVC pipe, semipermeable board, positioning plate, water level controller, water inlet pipe, drainage pipe, and sampling device, as illustrated in Figure 1a. The tank body is a rectangular block sealed using acrylic plates, with support rods inside to prevent deformation, semipermeable baffles at both ends, positioning plates, and water level controllers outside. The simulated liquid storage tank and the waste liquid collection bucket are connected through the water inlet and drainage pipes. A total of 25 sampling holes are located on the front, and a simulated pumping well is installed. Please refer to the Supporting Information (Text S1 and Figure S1) for detailed information on the sand tank structure. The hematite sand column was filled with quartz sand, clay, and a hematite lens body, as illustrated in Figure 1b. Based on a profile of an actual aquifer structure from the field, different particle sizes of quartz sand (from 50 to 200 mesh) and clay were proportionally and sequentially loaded into the sand tank. The column was set to a height of approximately 30 cm, with the first 21 cm consisting of sand layers, followed by a clay layer from 21 to 29 cm and large quartz particles from 29 to 30 cm to prevent the clay from floating during the experiment.

Before the experiment, the flow rate of the peristaltic pump for the water supply was adjusted to 2.0 mL/min, and the flow rate of the peristaltic pump for the water extraction was adjusted to 1.0 mL/min. As shown on the left side of Figure 1c, the water level at the inlet is 3–4 mm higher than that at the outlet; this provides a horizontal hydraulic gradient of 4.6 × 10^−3^ to 6.2 × 10^−3^. The arsenic-containing simulated fluid is pumped into the system, and the liquid level in the tank gradually increases until it maintains an approximate 2 cm free water layer (top layer) during the experiment. The channel is stabilized for 5 days to achieve local chemical equilibrium between the minerals and the injected water. During the pumping phase, the liquid is drawn from the bottom sand layer at a 1.0 mL/min rate through the “well”, driving the oxidized water at the top to flow downward through the heterogeneous layer system. The water supply rate at the inlet exceeds the pumping rate at the well; this causes excess water to flow directly over the top water layer toward the outlet side rather than seep into the quartz sand medium.

### 2.2. Reagents and Samples

In the experiment, quartz sand was used as the reaction medium and underwent multiple cycles of soaking and rinsing with deionized water until the conductivity reached a stable value of approximately 30 μS/cm. Afterwards, the sand was subjected to drying in a forced-air oven at a temperature of 50 °C and was then set aside for future utilization. Hematite was purchased from the Hubei Exi Geological Industry and Trade Co., Hubei, China Ltd., Shiyan, China. The experimental water was ultrapure (conductivity < 18 μS/cm) and was used to prepare a 10.9203 mg/L Na_2_HAsO_4_·7H_2_O simulation solution.

The experiment utilized analytical quality reagents, including Na_2_HAsO_4_·7H_2_O, NaAsO_2_, H_2_NCSNH_2_, and C_6_H_8_O_6_. Additionally, KOH, NaOH, and HCl were used. The 1000 μg/mL arsenic standard solution was acquired from the National Nonferrous Metals and Electronic Materials Analysis and Testing Center. Analytical-grade reagents were used.

### 2.3. Tests and Methods

The concentrations of As(V) in the solution were measured using atomic fluorescence spectroscopy (AFS-933/SA-20, Beijing Jitian Instruments, Beijing, China; detection limit ≤ 0.01 mg/L). The elemental lamp used was an atomic fluorescence hollow cathode lamp (HAF-2) with a current of 36 mA. The dissolved iron (Fe(aq)) concentrations were determined using a microplate reader (SPARK, TECAN, Männedorf, Switzerland).

The main phases and crystallinity of the quartz sand samples were analyzed using an X’Pert3 Powder multifunctional X-ray diffractometer (PANalytical, BV, The Netherlands, Cu target, λ = 1.54056 Å). The scan step was set at 0.026°, with a scanning speed of 0.65(°)/s and a range from 5° to 90°. Fourier transform infrared spectroscopy (FTIR, iS 10, Thermo Fisher Scientific, Waltham, MA, USA) was used to determine the functional groups and chemical bond vibrations. The surface morphology and elemental composition were examined using scanning electron microscopy and energy dispersive spectroscopy (SEM-EDS, JSM-7900F, JEOL Ltd., Tokyo, Japan). The composition of the arsenic bound to the surface of the quartz sand was analyzed using an improved Wenzel extraction method [24], as shown in Table 1.

### 2.4. Statistical Analysis and Plotting Software

All data were statistically analyzed using Excel 2016, and all figures were generated using Origin 2021.

## 3. Results and Discussion

### 3.1. Migration Characteristics of As

**Figure 2:**
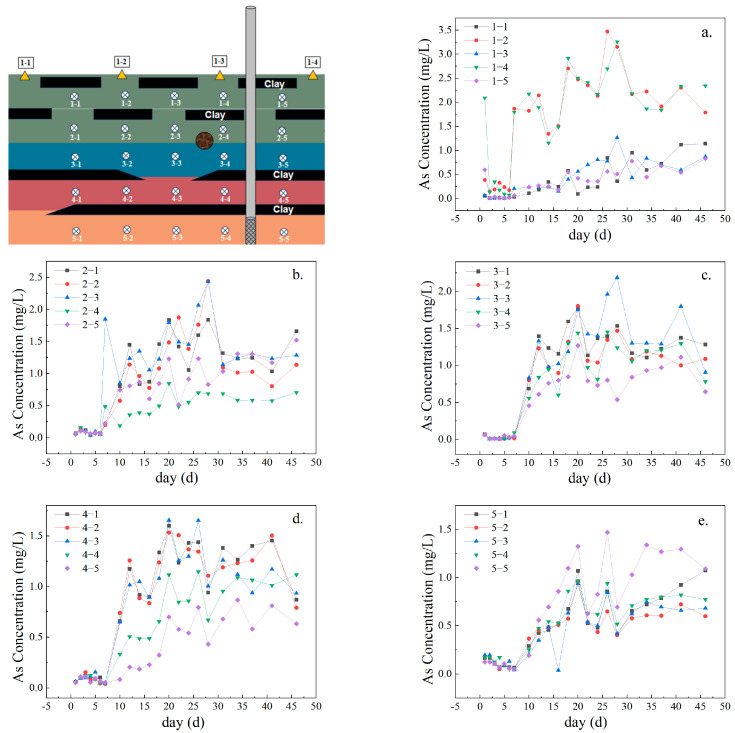
As(V) migration curve. (**a**) Arsenic concentration changes in layer one; (**b**) Arsenic concentration changes in layer two; (**c**) Arsenic concentration changes in layer three; (**d**) Arsenic concentration changes in layer four; (**e**) Arsenic concentration changes in layer five.

Figure 2a shows that the As(V) concentrations in sections 1-2 and 1-4 are significantly higher than those in sections 1-1, 1-3, and 1-5. As shown in Figure 2b, the concentrations in the second layer at points 2-1, 2-2, and 2-3 are notably higher. These results are in agreement with existing research; this research has revealed that the clay layers are impermeable, leading to higher flow rates in more permeable quartz sand layers and the formation of preferential flow channels [25]. Thus, by forming preferential flow pathways, the clay layer facilitates the rapid migration of As(V) in samples 1-2, 1-4, 2-1, 2-2, and 2-3.

Figure 2c,d show that the As(V) concentrations are significantly greater in the third layer at points 3-1, 3-2, and 3-3 and in the fourth layer at points 4-1, 4-2, and 4-3. These results indicate that the clay within the sand layers significantly affects the migration of As(V) in the third and fourth layers; thus, under the vertical pressure induced by pumping, the clay layers force As(V) to migrate horizontally. Duan [15] and Close [26] also reported that clay layers without hematite promoted the horizontal migration of As(V). However, their sampling points with the highest concentrations differed from ours; their highest concentrations were found at points 3-4, 3-5, 4-4, and 4-5. These results indicate that the hematite lens bodies influenced As(V) migration at locations 3-4 and 3-5, primarily through the dissolution and release of hematite and the formation of iron–aluminum oxide colloids. As water flows, hematite gradually dissolves and continuously releases Fe(aq) into the water. This released Fe(aq) is a key source of iron–aluminum oxides and contributes to the formation of new iron–aluminum oxides that adsorb As(V). This resulted in significantly lower As(V) concentrations at locations 3-4 and 3-5 than at the other sampling sites.

Figure 2e illustrates the migration pattern of As(V) in the fifth-layer medium. As pumping progresses, the As(V) concentrations are similar across most sampling points, except for a higher concentration at sample points 5-5. These results align with the studies by Duan [15] and Qin [27]; their studies indicated that the distribution of As(V) was relatively uniform in the bottom layer, with rapid horizontal flow and stable flow rates. This result also shows that the distribution of As(V) is relatively uniform in relatively closed groundwater spaces.

Notably, the equilibrium concentrations of As(V) in each layer are inversely proportional to depth. As the depth increases, the maximum concentration of As(V) gradually decreases from 3.5 mg/L to 1.5 mg/L. Thus, the As distribution in heterogeneous media is highly uneven, indicating that the low permeability of clay layers impedes the diffusion and migration of As(V). Based on a comparison of the changes in As(V) concentrations at different depths between days 10 and 15, the As concentrations in various media layers decreased to varying extents. The most significant decreases were in the first and fourth layers, which decreased from 2.3 mg/L and 1.3 mg/L to 1.2 mg/L and 0.82 mg/L, respectively. These results indicate that diffusion cannot solely explain the migration of As(V) in the heterogeneous sand column. Adsorption by Fe(aq) and quartz sand also plays a crucial role in controlling the movement of As(V). The content of As(III) remains extremely low, and its concentration shows minimal fluctuation, indicating that the change in total As concentration is primarily due to As(V). 

### 3.2. Fe Ion Release and Migration Characteristics

**Figure 3:**
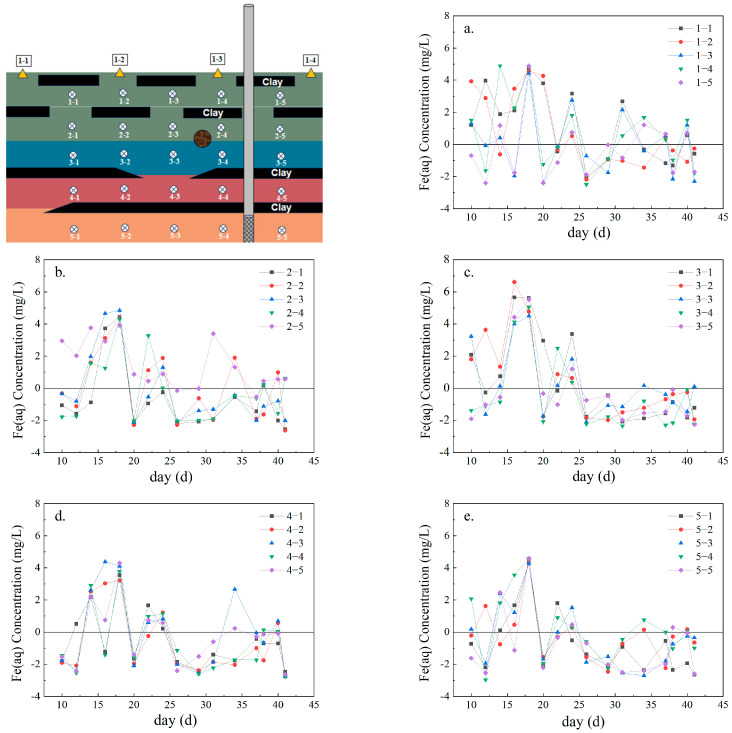
Fe(aq) migration curve. (**a**) Change of Fe(aq) concentration in the first layer; (**b**) Change of Fe(aq) concentration in the second layer; (**c**) Change of Fe(aq) concentration in the third layer; (**d**) Change of Fe(aq) concentration in the fourth layer; (**e**) Change of Fe(aq) concentration in the fifth layer.

As shown in Figure 3, during the test period, the concentration of Fe(aq) in the surface layer (1-2 layers) minimally changed, and after 40 days of pumping, the concentration of Fe(aq) remained above 0.65 mg/L. Figure 3a,b show that the surface concentration of Fe(aq) reaches a maximum on day 18; thus, pumping creates preferential flow paths, and Fe(aq) diffuses against the direction of pumping along these pathways [28]. Previous research has also shown that extracting high-arsenic groundwater for irrigation activities causes groundwater levels to rise, promoting the reductive dissolution of iron oxides [29,30,31]. Therefore, from 0 to 18 days, pumping promotes the reductive dissolution of hematite, leading to a rapid increase in the concentration of Fe(aq).

Figure 3c,d show that the Fe(aq) concentration rapidly increases to its maximum before day 16 and then decreases to 0 mg/L after 25 days. Existing research indicates that the release process of minerals such as hematite involves two stages: a rapid dissolution and release phase followed by a dissolution equilibrium phase [32,33]. Thus, hematite is initially released quickly and then reaches concentration equilibrium later. Although Figure 3c,d also show two stages, unlike existing studies, the concentration of Fe(aq) does not remain stable after rapid growth but quickly decreases. Moreover, the peak concentration of Fe(aq) in layer 3 is much higher than that in layer 4, indicating that water pumping can cause only part of the Fe(aq) to diffuse downward. In summary, although pumping leads to a rapid release of hematite, once it reaches the release equilibrium phase, Fe(aq) participates in the adsorption process of As(V), resulting in a rapid decrease in Fe(aq) levels.

In addition, as shown in Figure 3a,b,e, the maximum Fe(aq) concentrations in layers 1, 2, and 5 all appeared after 18 days; the amount of time needed to reach this maximum was significantly longer with respect to that in layers 3 and 4 (their maximum Fe(aq) concentrations were reached at 16 days). Previous studies have shown that after pollutants (as solutes) enter the underground saturated zone, they mainly exist in the groundwater in the form of ions and dissolved substances and migrate with the groundwater through advection and diffusion [34,35]. However, due to the low permeability of the clay layers, they are often considered impermeable in field studies. In layers 1, 2, and 5, the clay layer barrier effect restricted the solution’s advection and diffusion, hindering the migration of Fe(aq). Thus, the time to reach the maximum concentrations of Fe(aq) in these layers was extended.

### 3.3. Morphology and Distribution Characteristics of Solid As

**Figure 4:**
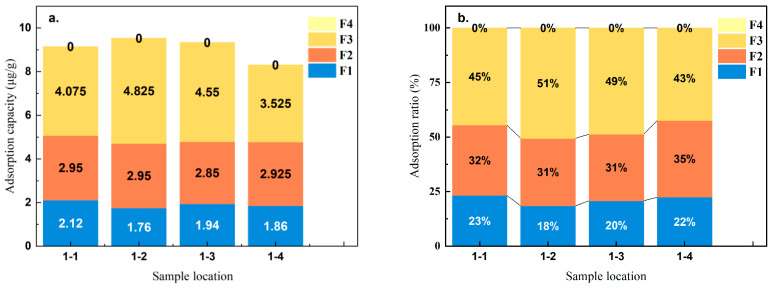
Contents and percentages of arsenic in its different adsorbed states. (**a**) Amount of arsenic adsorbed in each adsorbed state (μg/g); (**b**) Proportion of arsenic in each adsorbed state (%).

Figure 4a displays the total adsorption of As. The results show that the adsorption levels at points 1-2 and 1-3 are significantly greater than those at points 1-1 and 1-4, and the order is as follows: 1-2 > 1-3 > 1-1 > 1-4. Moreover, the highest content of As in the F3 binding state is found at points 1-2 and 1-3. A comparison of these sampling locations at points 1-2 and 1-3, which are located on the preferential flow channels, shows greater As adsorption. These findings highlight the importance of the preferential flow channels in enhancing As adsorption.

To further analyze the mechanism by which Fe(aq) adsorbs As, we compared the content of As in different binding states. Figure 4b shows that the predominant forms of As(V) adsorbed by quartz sand are F2 and F3, which together account for 77% to 82% of the total As. Thus, quartz sand primarily adsorbs As(V) as amorphous and weakly crystalline iron–aluminum oxides. Evidence from prior studies indicates that hematite primarily adsorbs As in the F1 and F2 forms [36,37] rather than in the F2 and F3 forms. This difference indicates that Fe(aq) no longer adsorbs As(V) on quartz sand surfaces in the form of hematite after the hematite dissolves. Zhong [38] reported that adding hematite to arsenic-containing soil significantly increased As adsorption in the F3 binding state.

Additionally, the oxidation of iron with atmospheric oxygen and the subsequent precipitation of iron minerals can cause a decrease in the As concentrations [39]. This result indicates that after hematite dissolves and releases Fe(aq), Fe(aq) forms hematite on the surface of quartz sand, further adsorbing As(V). Thus, weakly crystalline iron–aluminum oxides are the primary forms for As adsorption. Therefore, hematite can facilitate the adsorption of As onto quartz sand surfaces in the form of weakly crystalline iron–aluminum oxides [40,41].

Notably, Figure 4 shows no presence of crystalline iron–aluminum oxide-bound As (F4) at any of the sampling points from 1-1 to 1-4. This means that Fe(aq) cannot directly form crystalline iron oxides. Additionally, the As contents in samples 1-2 and 1-3 (9.535 μg/g and 9.340 μg/g) are significantly greater than those in samples 1-1 and 1-4 (9.145 μg/g and 8.310 μg/g). However, the proportions and amounts of F1 and F2 in samples 1-2 and 1-3 are lower than those in samples 1-1 and 1-4. Zhong’s research [38] showed that adding limonite and hematite to arsenic-containing soil did not significantly change the F4 content in the soil after the reaction. However, the F1 content decreased to varying degrees. Similarly, studies have shown that weakly crystalline iron minerals could effectively adsorb As [42,43,44]. Based on these results and studies, iron minerals adsorb As mainly by dissolving to form amorphous Fe-Al oxides. Zheying [45] reported that when the Fe(aq) concentration was lower than 1 mg/L, the adsorption of As by quartz sand was inhibited. After the dissolution of hematite released Fe(aq), Fe(aq) inhibited the adsorption of F1 and F2 while promoting the adsorption of F3. Thus, although some Fe(aq) was directly adsorbed by quartz sand at lower concentrations and occupied the F1 nonspecific adsorption sites, the dissolution of hematite released more Fe(aq). The quartz sand then adsorbed this excess Fe(aq), forming amorphous iron–aluminum oxides, which greatly increased the As(V) adsorption by the quartz sand.

## 4. Reaction Mechanism Analysis

### 4.1. Surface Morphology and Element Distribution

**Figure 5:**
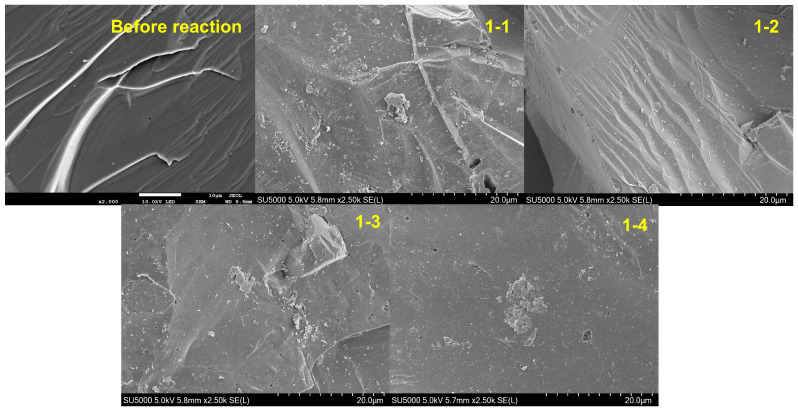
Microscopic morphology of sand before and after reaction.

As shown in Figure 5, the surface of the quartz sand was relatively smooth, with few particles present before the reaction. After the experiment, the surface of sample 1-1 contained numerous particles and mineral crystals, along with a few very small pores. Samples 1-2 and 1-3 also contain a lot of particulate matter. It should be noted that the surface of sample 1-2 exhibits ridge-like structures, with particles uniformly distributed along these ridges. The surface of sample 1-3 contained microporous structures, with some particles aggregating into more significant crystalline substances. The surface of sample 1-4 contained uniformly distributed particles and a few microporous structures.

Additionally, some particles had aggregated into moss-like crystalline substances. These results were consistent with those from Du [46]; in their study, the surface of river sand exhibited numerous grooves and flaky structures after adsorbing As. Other studies have also revealed that the surface of quartz sand had pore structures [47], and these pore structures facilitated the adsorption of As. Clearly, the surface of the quartz sand had few impurities before the reaction. Thus, the crystalline substances on the quartz sand surface after the reaction were likely to be iron and aluminum oxides.

**Figure 6:**
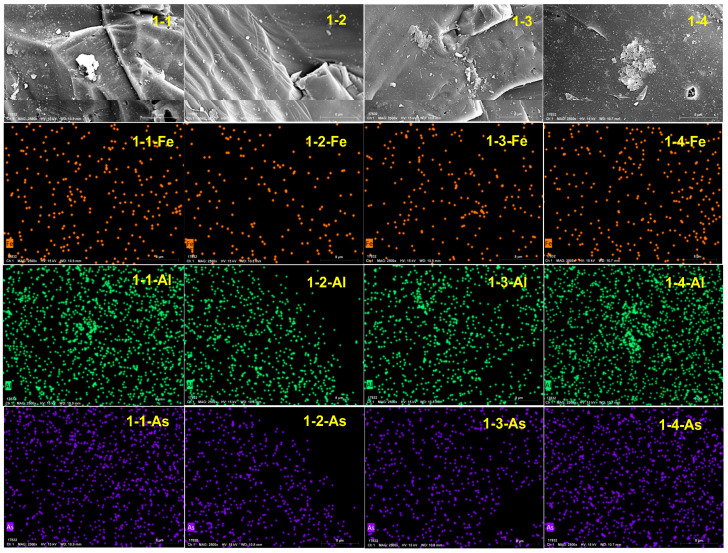
Surface energy spectrum of sand before and after the reaction.

To further investigate the composition of the crystalline substances on the quartz sand surface after the reaction, EDS was performed. As shown in Figure 6, while Al is enriched, the Fe and As are relatively dispersed. Additionally, a comparison of the distribution of Al reveals that it is concentrated in the mineral crystals, indicating that the crystals on the quartz sand surface are aluminum oxides. Additionally, no As enrichment is observed at the locations where aluminum is concentrated, indicating that aluminum oxides on the quartz sand surface do not effectively adsorb As. Thus, the Fe (hydro)oxides on the quartz sand surface provide adsorption sites for As [48,49], enhancing its adsorption.

Based on the data in Table 2, minimal amounts of iron oxides are effective for arsenic adsorption in samples 1-2 and 1-3. This demonstrates the ability of iron oxides to bind arsenic efficiently. Conversely, no arsenic was detected in samples 1-1 and 1-4, where iron oxides are absent. Notably, a comparison of the percentages of Fe and As on the surfaces of samples 1-2 and 1-3 reveals significantly lower Fe (0.08) and As (0.01) contents in sample 1-2 with respect to Fe (0.31) and As (0.02) in sample 1-3. These results indicate that the adsorption capacity of river sand for As increases after Fe (hydro)oxides attach to the surface of quartz sand. Thus, Fe (hydro)oxides can effectively enhance the adsorption of As [50].

### 4.2. Main Crystalline Phase on the Surface

**Figure 7:**
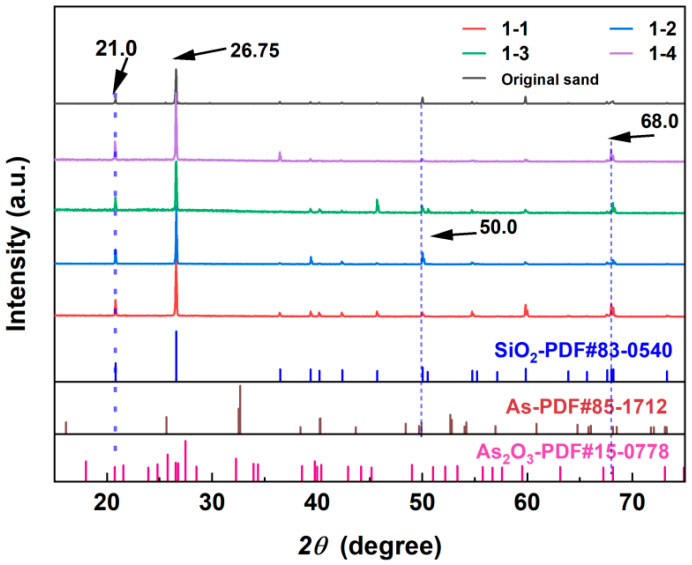
Physical phase of sand after reaction.

The XRD patterns of the quartz samples before and after the reaction at different locations are shown in Figure 7. The results indicate that the main component of the river sand surface was SiO_2_. Trace amounts of As compounds were detected on the surface of the quartz sand. After the pumping test, the quartz sand spectra revealed several distinct characteristic peaks. The peak at 2θ = 26.75° corresponded to SiO_2_, indicating that SiO_2_ was the dominant component in all samples. Based on previous studies, regardless of the presence of organic matter in riparian sediments, the main phase detected by XRD after the adsorption reaction remained SiO_2_ [51]. After the pumping test reached equilibrium, the peak intensity at 2θ = 21° in the XRD pattern slightly increased. This was potentially caused by As adsorbing onto the quartz sand surface as As_2_O_3_. The peak intensity at 2θ = 68° significantly increased. Compared with standard reference cards (PDF85-1712 and PDF15-0778), this was likely the result of the combined effects of As and As_2_O_3_. The peak intensity of 1-2 at 2θ = 50° significantly increased. Compared with the standard card (PDF85-1712), the As on the surface of the quartz sand mainly existed in the form of an As acid salt [52].

### 4.3. Changes in the Surface Functional Groups

**Figure 8:**
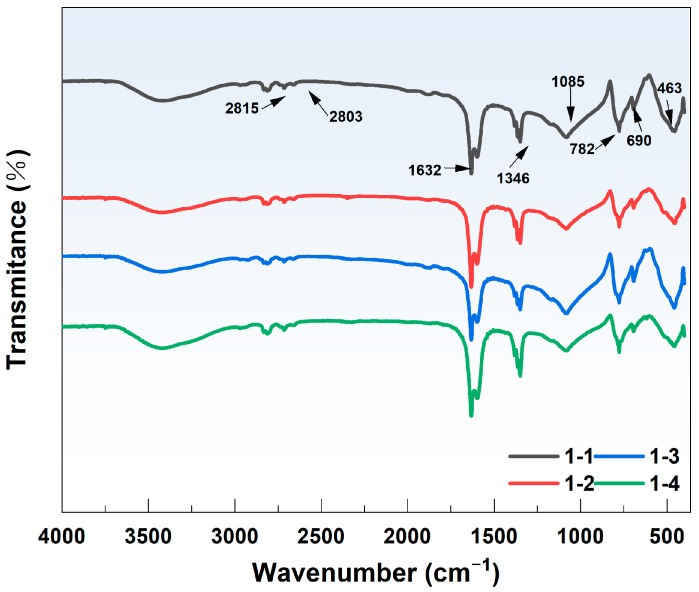
Crown group of sand after reaction.

The FTIR spectra of the quartz sand after the reaction at different locations are shown in Figure 8. No significant differences were observed between the FTIR spectra of quartz sand before and after As adsorption at the different locations. Under high-concentration conditions (Fe(aq) = 20 mg/L), a characteristic absorption peak for SiO_2_ appears at 690 cm^−1^ [53]. Thus, the main component is SiO_2_; this result is consistent with the results from the EDS and XRD analyses. Previous studies have shown that characteristic vibrational absorption peaks of Si–O are present at 463.06, 797.49, and 1084.72 cm^−1^ [54]. Si–O bond stretching vibrations cause peaks at 463.06 and 797.49 cm^−1^, whereas the peak at 1084.72 cm^−1^ is due to the antisymmetric stretching vibration of Si–O–Si [45]. The characteristic absorption peaks observed between 950 and 1250 cm^−1^ are attributed primarily to the stretching vibrations of Si–OH and the antisymmetric stretching vibrations of Si–O–Si [55]. After the reaction, the surface of the quartz sand did not show the characteristic peaks of Fe minerals such as α-FeOOH (889, 795 cm^−1^) [56], γ-FeOOH (1020 cm^−1^) [57], Fe_2_O_3_ (559, 427 cm^−1^) [58], and Fe_3_O_4_ (586 cm^−1^) [59]. These results indicate that no new iron-containing functional groups were formed during the reaction. In addition, under low-concentration conditions (Fe(aq) = 0.1 mg/L), characteristic absorption peaks of aldehydes were found at 2815 and 2803 cm^−1^ in all samples. The stretching vibrations of the C–H bond caused these peaks. The 1632 and 1346 cm^−1^ peaks were attributed to the C=O stretching vibrations and –OH bending vibrations, respectively. These absorption peaks were formed by a small number of water molecules [60].

## 5. Conclusions

In this study, the migration and distribution behaviors of As(V) and aqueous iron (Fe(aq)) in a groundwater environment were examined using a sand column experiment embedded with hematite lenses. Solid-phase extraction and characterization analysis was applied to investigate the control mechanisms of the As(V) migration. Our study reveals the intricate dynamics of As migration and distribution within different geological substrates, highlighting the role of the medium structure, particularly the arrangement of clay layers and quartz sand. These substrates distinctly affected the As concentrations. In the case of hematite, dissolution initially occurred at a rapid pace, and Fe(aq) was released into the system. However, this release rate notably diminished once a certain concentration threshold was reached, showing a crucial factor in the As distribution. Furthermore, our findings on solid-phase As indicated varying binding states, and that an amorphous iron–aluminum oxide-bound state and specifically adsorbed state were predominant under the influence of preferential flow channels, which facilitated the enhanced As adsorption. Hematite dissolution releases Fe(aq). Fe(aq) subsequently reduces the migration of As(V) by modifying its adsorption and binding on quartz sand. These insights are critical for developing robust strategies for groundwater As pollution management, providing a theoretical foundation for the development of effective As mitigation approaches.

## Figures and Tables

**Table 1:** Wenzel extraction method.

Chemical Form	Extraction Method	Water–Sand Ratio (mL:g)
Nonspecifically adsorbed state (F1)	0.05 mol/L (NH4)_2_SO_4_, 25 °C, shaking 4 h	25:1
Specifically adsorbed state (F2)	0.05 mol/L NH_4_H_2_PO_4_, 25 °C, shaking 16 h	25:1
Amorphous iron-aluminum oxide-bound state (F3)	First time: 0.2 mol/L ammonium oxalate buffer (pH = 3.5), 25 °C, shaking 4 h Second time: 0.2 mol/L ammonium oxalate buffer (pH = 3.5), 25 °C, shaking 10 min	First time 25:1 Second time 12.5:1
Crystalline iron-aluminum oxide-bound state (F4)	First time: ammonium oxalate (0.2 mol/L) + ascorbic acid (0.1 mol/L), pH = 3.5, 96 °C, shaking 30 min Second time: ammonium oxalate (0.2 mol/L) + ascorbic acid (0.1 mol/L), pH = 3.5, 96 °C, shaking 10 min	First time 25:1 Second time 12.5:1

**Table 2:** Elemental contents of sand before and after reaction with As and Fe (unit: %).

Condition	C	O	Si	Al	Fe	As
Before reaction	4.47	52.14	42.45	0.21	0.73	0
1-1	5.97	41.66	52.13	0.23	0	0
1-2	4.81	44.49	50.05	0.04	0.08	0.01
1-3	5.07	38.89	55.63	0.07	0.31	0.02
1-4	6.38	43.30	50.25	0.07	0	0

## Data Availability

The original contributions presented in the study are included in the article material, further inquiries can be directed to the corresponding authors.

## Supplementary Materials

The following supporting information can be downloaded at: https://www.mdpi.com/article/10.3390/toxics12090687/s1, Figure S1: Sand tank structure diagram; Table S1: Chemical compositions of medium (%). Text S1: Detailed information for sand tank.

## References

[1] Podgorski J., Berg M. (2020). Global threat of arsenic in groundwater. Science.

[2] Cao W., Fu Y., Cheng Y., Zhai W., Sun X., Ren Y., Pan D. (2023). Modeling potential arsenic enrichment and distribution using stacking ensemble learning in the lower Yellow River Plain, China. J. Hydrol..

[3] WHO (World Health Organization) (2017). Guidelines for Drinking Water Quality: Fourth Edition Incorporating the First Addendum.

[4] Guo J., Cao W., Lang G., Sun Q., Nan T., Li X., Ren Y., Li Z. (2024). Worldwide Distribution, Health Risk, Treatment Technology, and Development Tendency of Geogenic High-Arsenic Groundwater. Water.

[5] Sinha D., Prasad P. (2020). Health effects inflicted by chronic low-level arsenic contamination in groundwater: A global public health challenge. J. Appl. Toxicol..

[6] Xu N., Zhang F., Xu N., Li L., Liu L. (2023). Chemical and mineralogical variability of sediment in a quaternary aquifer from Huaihe River Basin, China: Implications for groundwater arsenic source and its mobilization. Sci. Total Environ..

[7] Yuan R., Li Z., Guo S. (2023). Health risks of shallow groundwater in the five basins of Shanxi, China: Geographical, geological and human activity roles. Environ. Pollut..

[8] Biswas T., Pal S.C., Saha A., Ruidas D. (2023). Arsenic and fluoride exposure in drinking water caused human health risks in coastal groundwater aquifers. Environ. Res..

[9] McArthur J.M., Nath B., Banerjee D.M., Purohit R., Grassinea N. (2011). Palaeosol control on groundwater flow and pollutant distribution: The example of arsenic. Environ. Sci. Technol..

[10] Brikowski T.H., Neku A., Shrestha S.D., Smith L.S. (2014). Hydrologic control of temporal variability in groundwater arsenic on the Ganges floodplain of Nepal. J. Hydrol..

[11] Wang J., Li Z., Zhu Q., Wang C., Tang X. (2023). Review on arsenic environment behaviors in aqueous solution and soil. Chemosphere.

[12] Fischel M.H.H., Clarke C.E., Sparks D.L. (2024). Arsenic sorption and oxidation by natural manganese-oxide-enriched soils: Reaction kinetics respond to varying environmental conditions. Geoderma.

[13] Li C., Bundschuh J., Gao X., Li Y., Zhang X., Luo W., Pan Z. (2022). Occurrence and behavior of arsenic in groundwater-aquifer system of irrigated areas. Sci. Total Environ..

[14] Cheng H., Hu Y., Luo J., Xu B., Zhao J. (2009). Geochemical processes controlling fate and transport of arsenic in acid mine drainage (AMD) and natural systems. J. Hazard. Mater..

[15] Duan Y., Li R., Gan Y., Yu K., Tong J., Zeng G., Ke D., Wu W., Liu C. (2020). Impact of physico-chemical heterogeneity on arsenic sorption and reactive transport underwater extraction. Environ. Sci. Technol..

[16] Duan Y., Li R., Yu K., Zeng G., Liu C. (2022). Effects of geochemical and hydrodynamic transiency on desorption and transport of As in heterogeneous systems. Sci. Total Environ..

[17] Zou Q., Wei H., Chen Z., Ye P., Zhang J., Sun M., Huang L., Li J. (2023). Soil particle size fractions affect arsenic (As) release and speciation: Insights into dissolved organic matter and functional genes. J. Hazard. Mater..

[18] Aftabtalab A., Rinklebe J., Shaheen S.M., Niazi N.K., Moreno-Jiménez E., Schaller J., Knorr K.H. (2022). Review on the interactions of arsenic, iron (oxy)(hydr) oxides, and dissolved organic matter in soils, sediments, and groundwater in a ternary system. Chemosphere.

[19] Cai X., Yin N., Liu X., Wang P., Du H., Cui Y., Hu Z. (2022). Biogeochemical processes of arsenic transformation and redistribution in contaminated soils: Combined effects of iron, sulfur, and organic matter. Geoderma.

[20] Lu S., Su X., Feng X., Sun C. (2022). Study on the formation and influencing factors of arsenic in nearshore groundwater during river water infiltration. Earth Sci. Front..

[21] Zhang D., Guo H., Xiu W., Ni P., Zheng H., Wei C. (2017). In-situ mobilization and transformation of iron oxides-adsorbed arsenate in natural groundwater. J. Hazard. Mater..

[22] Nath B., Berner Z., Mallik S.B., Chatterjee D., Charlet L., Stueben D. (2005). Characterization of aquifers conducting groundwaters with low and high arsenic concentrations: A comparative case study from West Bengal, India. Mineral. Mag..

[23] Fendorf S., Michael H.A., Van Geen A. (2010). Spatial and temporal variations of groundwater arsenic in South and Southeast Asia. Science.

[24] Wenzel W.W., Kirchbaumer N., Prohaska T., Stingeder G., Lombi E., Adriano D.C. (2001). Arsenic fractionation in soils using an improved sequential extraction procedure. Anal. Chim. Acta.

[25] Shepley M.G., Schmidt N. (2022). Utility trench water level recessions in an aquitard: Findings from analytical and numerical analyses. Hydrogeol. J..

[26] Glose T.J., Zipper S., Hyndman D.W., Stingeder G., Lombi E., Adriano D.C. (2022). Quantifying the impact of lagged hydrological responses on the effectiveness of groundwater conservation. Water Resour. Res..

[27] Qin R., Wu Y., Xu Z., Xie D., Zhang C. (2013). Numerical modeling of contaminant transport in a stratified heterogeneous aquifer with dipping anisotropy. Hydrogeol. J..

[28] Wu Q., Zhang J., Lin W., Wang G. (2014). Dye tracing of soil water flow pattern and evaluation of preferential flow degree. Trans. Chin. Soc. Agric. Eng..

[29] Yan Y., Xie X., Zheng W., Chi Z., Liu Y. (2017). Effects of irrigation activities on arsenic migration in surface soil of Datong Basin. Geol. Sci. Technol. Inf..

[30] Li H., Ding S., Song W., Wang X., Ding J., Lu J. (2022). The degradation of dissolved organic matter in black and odorous water by humic substance-mediated Fe (II)/Fe (III) cycle under redox fluctuation. J. Environ. Manag..

[31] Xu F., Li P. (2024). Biogeochemical mechanisms of iron (Fe) and manganese (Mn) in groundwater and soil profiles in the Zhongning section of the Weining Plain (northwest China). Sci. Total Environ..

[32] Zhang Q., Wang X., Chen J., Zhuang G. (2006). Heterogeneous reaction mechanism of SO_2_ and Fe_2_O_3_ to form Fe(II)(aq) and sulfate. J. Chem. Univ..

[33] Peng X., Yang B., Li X., Dai X., Wei C., Lu Z., Deng Z., Li M., Fan G. (2024). Dissolution behavior of hematite in H_2_SO_4_ solution: A kinetic analysis and its importance on the zinc hydrometallurgical hematite process. Miner. Eng..

[34] Zeng J., Tabelin C.B., Gao W., Tang L., Luo X., Ke W., Jiang J., Xue S. (2023). Heterogeneous distributions of heavy metals in the soil-groundwater system empowers the knowledge of the pollution migration at a smelting site. Chem. Eng. J..

[35] Yao Y., Mi N., He C., Yin L., Zhou D., Zhang Y., Sun H., Yang S., Li S., He H. (2020). Transport of arsenic loaded by ferric humate colloid in saturated porous media. Chemosphere.

[36] An L., Liu M., Zhang J., Huang L., Chen Z. (2020). Research progress on the sources of arsenic in soil and factors affecting its migration and release. Soils.

[37] Zhang Y., Xie X., Sun S., Wang Y. (2023). Arsenic transformation and redistribution in groundwater induced by the complex geochemical cycling of iron and sulfur. Sci. Total Environ..

[38] Zhong S., Yin G., He H., Huang R., Chen Z., Lin Q., Peng H., Wang K. (2017). Stabilization effect and mechanism of different iron minerals on arsenic in paddy soil. J. Environ. Sci..

[39] Park J.H., Han Y.S., Ahn J.S. (2016). Comparison of arsenic co-precipitation and adsorption by iron minerals and the mechanism of arsenic natural attenuation in a mine stream. Water Res..

[40] Yang Z., Zeng X., Sun B., Su S., Wang Y., Zhang N., Zhang Y., Wu C. (2021). Research progress on fixation of soil heavy metals by iron oxides. Soil Bull..

[41] Yao D., Shi Y., Pan H., Zhong D., Hou H., Wu X., Chen J., Wang L., Hu Y., Crittenden J.C. (2020). Promotion mechanism of natural clay colloids in the adsorption of arsenite on iron oxide particles in water. Chem. Eng. J..

[42] Inchaurrondo N., Di Luca C., Haure P., Žerjav G., Pintar A., Palet C. (2021). Evaluation of low-cost geo-adsorbents for As (V) removal. Environ. Technol. Innov..

[43] Wang H., Tsang Y.F., Wang Y., Sun Y., Zhang D., Pan X. (2018). Adsorption capacities of poorly crystalline Fe minerals for antimonate and arsenate removal from water: Adsorption properties and effects of environmental and chemical conditions. Clean Technol. Environ. Policy.

[44] Zhang D., Wu S., Wei Y., Zhou L. (2022). Schwertmannite modified with ethanol: A simple and feasible method for improving As (III) adsorption capacity. J. Environ. Chem. Eng..

[45] Li Z., Peng S., Shan H., Liao Q., Zhou H., Zhao Z. (2024). The Influence of Aqueous Iron on River Sand’s Arsenic Adsorption: Characteristics and Mechanisms. Water.

[46] Du H., Shan H., Huang J., Zeng C., Zhang X., Liu Y. (2024). Experimental study on the effects of flow velocity and medium particle size on As(III) migration. Earth Sci..

[47] Itamiya H., Sugita R., Sugai T. (2019). Analysis of the surface microtextures and morphologies of beach quartz grains in Japan and implications for provenance research. Prog. Earth Planet. Sci..

[48] Ding K., Ruan L., Wang H., Deng X., Yang W., Jiang N., Zhang J. (2024). Research progress on the application of FeOOH in adsorption of water pollutants. Appl. Chem. Ind..

[49] Zhang Y., Hou Z., Fu P., Wang X., Xue T., Chen Y. (2023). Simultaneous stabilization of arsenic and antimony co-contaminated mining soil by Fe(II) activated-Fenton sludge: Behavior and mechanisms. Environ. Pollut..

[50] Li J., Zhan M., Zhong X., Wang Y., Ou Y., Zhao X. (2021). Accumulation characteristics and influencing factors of heavy metals in soil-crop systems in typical karst areas of Guangxi. J. Environ. Sci..

[51] Nguyen K.T., Navidpour A.H., Ahmed M.B., Mojiri A., Huang Y., Zhou J.L. (2022). Adsorption and desorption behavior of arsenite and arsenate at river sediment-water interface. J. Environ. Manag..

[52] Hou Q., Zhang Y., Yu K., Han D., Chen J. (2023). Dynamic transformation of soil arsenic binding forms driven by flooding-drying cycles. Sci. Technol. Eng..

[53] Chen H.S., Sun Z.Y., Shao J.C. (2011). Investigation on FT-IR Spectroscopy for Eight Different Sources of SiO_2_. Bull. Chin. Ceram. Soc..

[54] Sun S.Y., Wen K., Yang B., Zhou Q., Dong F., Nie X., Liu L., Fan S. (2013). The preparation and adsorption properties of novel active carbon/diatomite. Acta Petrol. Mineral..

[55] Wang Y., Yu W., Chang Z., Gao C., Yang Y., Zhang B., Wang Y., Xing B. (2022). Effects of dissolved organic matter on the adsorption of norfloxacin on a sandy soil (fraction)from the Yellow River of Northern China. Sci. Total Environ..

[56] Zhang L., Song L.T., Zheng X.D., Teng Y., Wang J. (2014). The remobilization of heavy metals influenced by interaction of DOM and iron oxides. Chin. J. Ecol..

[57] Adames-Montero Y., López-Guerra S., Marrero-Águila R., Cueli-Corugedo A., Davis-Harriett J. (2020). Transformaciones físico-químicas de productos de corrosión el hierro en instalaciones petroleras. Tecnol. Química.

[58] Wang H.M., Ma Y.P., Chen X.Y., Xu S., Chen D., Zhang L., Zhao B., Ning P. (2020). Promoting effect of SO_4_^2−^ functionalization on the performance of Fe_2_O_3_ catalyst in the selective catalytic reduction of NOx with NH_3_. J. Fuel Chem. Technol..

[59] Zulfikar M.A., Utami A.R., Handayani N., Wahyuningrum D., Setiyanto H., Azis M.Y. (2022). Removal of phthalate ester compound from PVC plastic samples using magnetic molecularly imprinted polymer on the surface of superparamagnetic Fe_3_O_4_ (Fe_3_O_4_@ MIPs). Environ. Nanotechnol. Monit. Manag..

[60] Kefirov R., Ivanova E., Hadjiivanov K., Dzwigaj S., Che M. (2008). FTIR characterization of Fe^3+^–OH groups in Fe–H–BEA zeolite: Interaction with, CO and NO. Catal. Lett..

