# Supplementary material for: Characteristics and Mechanism of Hematite Dissolution and Release on Arsenic Migration in Heterogeneous Materials
Source: Toxics. 2024 Sep 23;12(9):687. doi: 10.3390/toxics12090687 (PMC11435827; doi:10.3390/toxics12090687)
Supplement: Supplementary file 1 [file toxics-12-00687-s001.zip › toxics-3189497-supplementary.pdf]

Text S1 and Figure S1

As shown in Figure S1.a, a sand tank device that can finely depict the migration and transformation of pollutants in an aquifer includes a tank body 1, a fixing rod 2, a PVC pipe 3, a semi-permeable board 8, a positioning board 9, a water level controller 4, a water inlet pipe 5, a drain pipe 6, a sampling hole 7, and a sampling device. The sand tank is rectangular and parallelepiped with an upper opening and a hollow interior sealed with acrylic plates. It is 60 cm long, 8 cm wide, and 40 cm high. The thickness of the organic glass is 0.7 cm. To prevent sand tank 1 from being compressed and deformed during the experiment, support rod 2 is connected to the middle of the sand tank 1 5 cm from the opening. The support rod 2 is an acrylic rod with a diameter of 1.5 cm. The support rod 2 and the sand tank 1 have threaded holes with a diameter of 0.7 cm on both sides. The support rod 2 is fixed to the sand tank 1 by metal screws.

Inside the sand, tank 1, a semi-permeable baffle 8 is provided 5 cm away from the left and right ends (sides in the width direction). The thickness of the semi-permeable baffle 8 is 1 cm, and the height is 35 cm. There is no hole in the lower 25 cm, and the upper 10 cm has uniform holes. During the experiment, it can effectively prevent the lateral flow of the water on the lower side and ensure the vertical flow. Positioning plates 9 are connected to the left and right ends of the sand tank 1. In this device, the positioning plate and the side of the sand tank (side in the length direction) are located in the same plane, and a longitudinal hole is opened 3 cm away from the sand tank 1. The length of the longitudinal hole is 35 cm, and the lateral width is 0.4 cm. As shown in Figure S1.b, a water level controller 4 is installed on the left and right ends of the sand tank. In this embodiment, the water level controller 4 is made of 0.7 cm thick organic glass and is a rectangular parallelepiped with an upper opening 7 cm long, 5 cm wide and 8 cm high. A 5 cm high baffle 12 is provided in the middle of the water level controller 4 in the width direction, which removes excess water during the water level adjustment process. Two water inlets, 10 and a water outlet, 11, are distributed on the lower side, connecting the sand tank, the simulated liquid storage tank and the waste liquid collection tank. The water level controller 4 controls the height through positioning plate 9 and is fixed on positioning plate 9 by friction through positioning hole 13 by plastic screws. The water level controller 4 and the sand tank 1 are connected through the water inlet pipe 5 and the drain pipe 6. In this device, the left end of the tank body is the water inlet end, and the right is the drain end. The water inlet pipe 5 is connected to the peristaltic pump, the simulated liquid storage tank is connected through the peristaltic pump tube, and drain pipe 6 is connected to the waste liquid collection bucket.

There are 25 sampling holes, 7 evenly distributed on the front side of the sand tank (the side in the length direction), and the sampling device is set at sampling hole 7, which includes a 22G needle 18, a silicone plug 19, and a three-way valve 17. As shown in Figure S1.c, the left side is the open state of the three-way valve, and the right side is the closed state of the three-way valve. The three-way valve consists of a water pumping port 14, a closing port 15 and a sampling port 16. The water pumping port 14 connects a syringe to extract the solution, the closing port 15 can be closed entirely, and the sampling port 16 is used to connect a needle to sample deep into the tank body. Figure S1.d is a schematic diagram of the sampling hole, and sampling hole 7 consists of a

three-way valve 17, a 22G needle 18 and a silicone plug 19. The sampling holes are arranged in 6 rows and 5 columns, with each row of sampling holes 7 separated by 5 cm and each column by 7.5 cm. The left and right columns of sampling hole 7 are separated by 6 cm from the semi-permeable plate 8, and the first row of sampling hole 7 at the lower end is separated by 5 cm from the bottom plate of the tank body 1.

A simulated pumping well 3 is also installed inside sand tank 1. The simulated pumping well 3 is a PVC pipe with an inner diameter of 1.3 cm. Only the lower end, with a length of 5 cm, is evenly opened, and water can enter. The opening position is wrapped with 300 mesh gauze to prevent the quartz sand from migrating due to pumping.

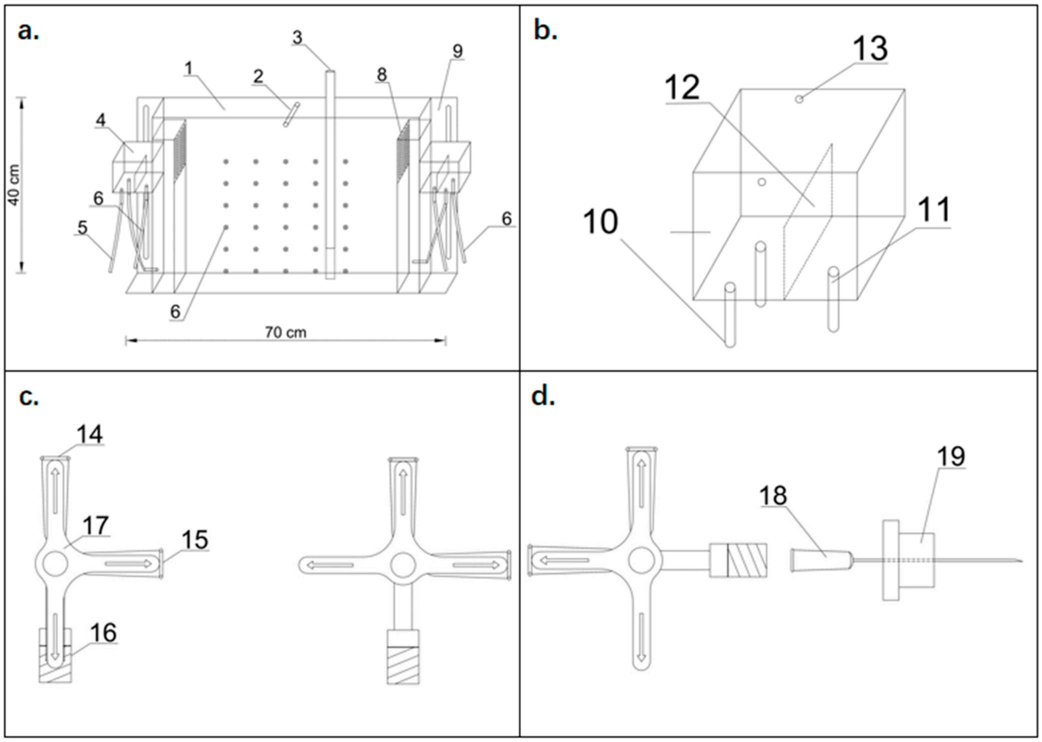

Figure S1 Sand tank structure diagram

Table S1

Table S1 Chemical compositions of medium (%).

Materials	SiO_2	CaO	Al_2O_3	MgO	Fe_2O_3	Na_2O	K_2O	TiO_2	P_2O_5
Quartz	94.28	-	4.29	-	0.156	-	0.799	0.15	-
Hematite	22.46	1.48	8.18	-	60.38	-	1.80	0.86	-
Clay	45.9	1.6	29.2	3.7	18.8	0.5	1.0	-	-
